# Following the Martensitic Configuration Footprints in the Transition Route of Ni-Mn-Ga Magnetic Shape Memory Films: Insight into the Role of Twin Boundaries and Interfaces

**DOI:** 10.3390/ma13092103

**Published:** 2020-05-01

**Authors:** Milad Takhsha Ghahfarokhi, Lucia Nasi, Francesca Casoli, Simone Fabbrici, Giovanna Trevisi, Riccardo Cabassi, Franca Albertini

**Affiliations:** Institute of Materials for Electronics and Magnetism, National Research Council (IMEM-CNR), Parco Area delle Scienze 37/A, 43124 Parma, Italy; milad.takhsha@imem.cnr.it (M.T.G.); lucia.nasi@imem.cnr.it (L.N.); francesca.casoli@imem.cnr.it (F.C.); simone.fabbrici@imem.cnr.it (S.F.); giovanna.trevisi@imem.cnr.it (G.T.); riccardo.cabassi@imem.cnr.it (R.C.)

**Keywords:** martensitic transition, Heusler alloys, magnetic shape memory alloys, twin boundary, epitaxial Ni-Mn-Ga films, transmission electron microscopy

## Abstract

Magnetic shape memory Heuslers have a great potential for their exploitation in next-generation cooling devices and actuating systems, due to their “giant” caloric and thermo/magnetomechanical effects arising from the combination of magnetic order and a martensitic transition. Thermal hysteresis, broad transition range, and twinning stress are among the major obstacles preventing the full exploitation of these materials in applications. Using Ni-Mn-Ga seven-modulated epitaxial thin films as a model system, we investigated the possible links between the phase transition and the details of the twin variants configuration in the martensitic phase. We explored the crystallographic relations between the martensitic variants from the atomic-scale to the micro-scale through high-resolution techniques and combined this information with the direct observation of the evolution of martensitic twin variants vs. temperature. Based on our multiscale investigation, we propose a route for the martensitic phase transition, in which the interfaces between different colonies of twins play the major role of initiators for both the forward and reverse phase transition. Linking the martensitic transition to the martensitic configuration sheds light onto the possible mechanisms influencing the transition and paves the way towards microstructure engineering for the full exploitation of shape memory Heuslers in different applications.

## 1. Introduction

Magnetic shape memory Heuslers provide new concepts for magnetic field-driven actuation [1,2], energy harvesting [3], and solid-state cooling technology [4] thanks to the magnetostructural martensitic phase transition and the giant magnetic field-induced strain, which has been reported in bulk single crystals as orders of magnitude higher than piezoelectric and magnetostrictive counterparts [5,6].

Ni-Mn-Ga films are a model system for magnetic shape memory materials. By exploiting epitaxial growth on different substrates, suitable growth conditions (including stress), and geometrical parameters (e.g., thickness) high quality and suitably oriented epitaxial films can be obtained. They allow accurate studies of structure and magnetism at the different length scales [7,8,9]. Despite their different martensitic configuration with respect to bulk materials, thin films also provide a suitable platform to study the role of intrinsic and extrinsic properties in the martensitic transformation process and formulate models that can be extended to bulk materials [10,11].

Being a type of thermoelastic material, epitaxial Ni-Mn-Ga films form interrelated 3D hierarchical patterns of twin boundaries in the martensitic phase to compensate the shear stress caused by the symmetry reduction during the martensitic phase transition from the cubic austenitic phase to the lower symmetry martensitic phase. The symmetry operator(s) for the twin boundaries connecting twin variants, is (are) rotation or (and) mirror [12,13,14,15]. The hierarchical nature of the originating from the self-accommodation occurring at the transition covers a broad range from nanoscale to macroscale [16].

For all the above-mentioned applications, the material cyclically undergoes one of the following phenomena.

(1) Martensitic phase transition induced by magnetic field, stress, or temperature.
(2) Magnetic field-induced reorientation of the twin variants in the martensitic phase.

The first phenomenon is based on the magnetostructural phase transition between the high-temperature austenitic and the low-temperature martensitic phases, while the latter only occurs in the low-temperature phase. It originates from the high magnetocrystalline anisotropy of the martensitic cells, which favors the alignment of the crystal cells in such a way that the magnetization easy axes of the cells are parallel to the external magnetic field [4]. It is well known that a few obstacles, stemming from both the intrinsic and extrinsic characteristics of the material, prevent the full exploitation of the aforementioned properties in applications. Among the obstacles are the thermal and magnetic hysteresis [17], broadness of the transition [18], and twinning stress [19], which are affected by several parameters, e.g., composition, chemical order, crystal structure, geometric compatibility of the martensite and austenite [20], dynamics of the transition [21], internal stress, and crystal defects. The two latter parameters are strongly linked to the configuration of the twin variants in the martensitic phase, which covers a long range from nano- to macro-scale [7,22,23]. 

Therefore, in order to find possible solutions to overcome these obstacles, it is necessary to gain a comprehensive view of the configuration of the twin variants at the different length scales and its evolution upon martensitic phase transition. In the literature, there are a few works focused on the crystallographic structures and the martensitic configurations of epitaxial Ni-Mn-Ga thin films through experiments and models [7,8,9,10,24,25,26]. However, the knowledge about the multiscale hierarchical self-accommodation of the twin variants in the martensitic phase and its possible links to the transition route is still limited, mainly due to lack of direct multiscale observation.

In this article, we report a multiscale study of martensitic Ni-Mn-Ga epitaxial films, characterized by a seven-fold incommensurate monoclinic superstructure with lattice modulation along the [001] crystallographic direction of the monoclinic setting [27]. In the martensitic phase, we directly visualize the crystallography of the epitaxial films, the symmetry relations between the twin boundaries and the interfaces between the different colonies of twin boundaries. We make use of various transmission electron microscopy (TEM) techniques with resolution from the atomic- to the micro-scale. This enables us to characterize the twin boundaries in a large-scale range. Our direct observations through TEM techniques in cross-sectional view are combined with atomic force microscopy (AFM) topography imaging vs. temperature. We propose a route for the martensitic forward and reverse transitions of the films, highlighting the major role played by the different martensitic interfaces.

## 2. Materials and Methods

### 2.1. Experimental

Epitaxial Ni-Mn-Ga films (200 nm) were prepared using radio frequency (RF) sputtering technique at elevated temperature (623 K) with a deposition rate of 0.1 nm s^−1^ and an Ar pressure of 1.5 Pa. The first sample (#1) was grown directly on (100) MgO followed by post-annealing (3600 s at 623 K in 10^−3^ mPa) and local mechanical stress (normal to the substrate, straining the film along the edge of MgO). The applied stress value was estimated to be >20 MPa, as described in [7], where complementary and detailed results on this sample can be found. The second sample (#2) was grown on 50 nm Cr underlayer, and in turn grown on the (100) MgO substrate. No post-growth treatment was performed for this sample. The composition measurement was performed using energy dispersive X-ray spectroscopy (EDS, EDAX detector, NJ, USA), obtaining Ni_52.7_Mn_19.9_Ga_27.4_, Ni_53.0_Mn_20.3_Ga_26.7_, for samples #1 and #2, respectively (uncertainty 1.0%). Atomic and magnetic force microscopy (Veeco Dimension 3100, CA, USA) imaging (supplementary material Figure S1) confirmed the X-type microstructure for the as-grown samples [26]. For sample #1, Y-type configuration stripes were induced by mechanical stress [7]. The details of X- and Y-microstructures are reported in Section 2.2. Different TEM techniques were performed with a JEOL 2200FS microscope (Tokyo, Japan) working at 200kV, i.e., high-angle angular dark-field scanning transmission electron microscopy (HAADF), high-resolution transmission electron microscopy (HR-TEM, Instrument), and selected-area electron diffraction (SAED). The lamellas for TEM observations were prepared parallel to [100] MgO (sample #1) and [1−10] MgO (sample #2) by focused ion beam lift-off technique using a Zeiss Auriga Compact scanning electron microscopy (Jena, Germany) equipped with Focused Ion Beam (FIB). Iso-field magnetic curves over temperature were measured by superconducting quantum interference device (SQUID) magnetometer (Quantum Design, CA, USA). In situ atomic force microscopy imagining versus temperature were measured by Dimension 3100 equipped with Nanoscope Veeco controller (Veeco Dimension 3100, CA, USA) using MESP-V2 tips.

### 2.2. Basic Concepts on the Crystallography of the Twin Boundaries

**Figure 1:**
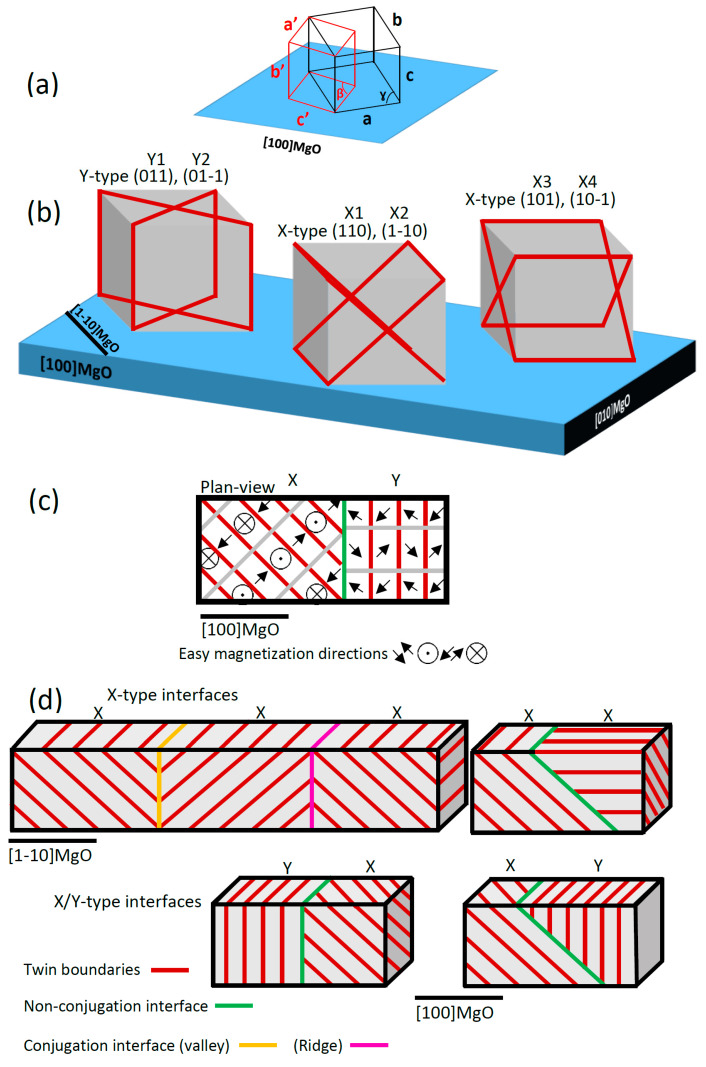
(**a**) Schematic representation of the relative orientations between the standard martensitic (red) and the austenitic setting (black) with respect to the MgO substrate directions. The indices show the three axes of the martensitic cell in the two represented settings. The martensitic seven modulation direction is along c’, whereas it is along one of the <110> directions in the austenitic setting. The easy magnetization direction in the two settings is b’ = c. The monoclinic non-right angle is β in the standard setting and γ in the austenitic setting; (**b**) Schematic representation of the {101} cubic planes in the Ni-Mn-Ga austenitic cell that are aligned with the twin boundaries in X-type and Y-type martensitic twin boundaries. (**c**) Top view of the X- and Y-type twin boundaries, showing the orientation of the magnetic easy axis in both the configurations. (**d**) Schematic representation of the martensitic interfaces in X-type and Y-type connecting the colonies of the twin boundaries nucleated from different twinning planes, color version is provided online.

Figure 1a shows the martensitic cell in the monoclinic (red) and the austenitic setting (black) with respect to the MgO substrate directions. The martensitic seven modulation direction (MD) is along c’ in the monoclinic setting, whereas it is along one of the <110> directions in the austenitic setting. The easy magnetization direction in the two settings is b’ = c. In epitaxial Ni-Mn-Ga films, differently from the bulk single crystals, the six {101} planes of the cubic austenitic cells are not equivalent. As shown in Figure 1b, the two planes which are normal to the substrate plane are closely aligned to the martensitic twin boundaries called Y-type, where the magnetic easy axis alternates in the plane of the film (Figure 1c). The four remaining {101} planes of the austenitic cells, which are 45° inclined with respect to the substrate, are closely aligned to the martensitic twin boundaries called X-type. For these configurations, the easy magnetization axis of the martensitic cell alternates in and out of the plane of the film (Figure 1c) [9]. As shown in Figure 1b, the six orientations of twin boundaries in X- and Y-type regions are conjugated in three pairs, i.e., Y1–Y2, X1–X2, and X3–X4, and give rise to a typical microstructure characterized by twin variants only oriented at 45° or at 90° with respect to the substrate plane. 

**Figure 2:**
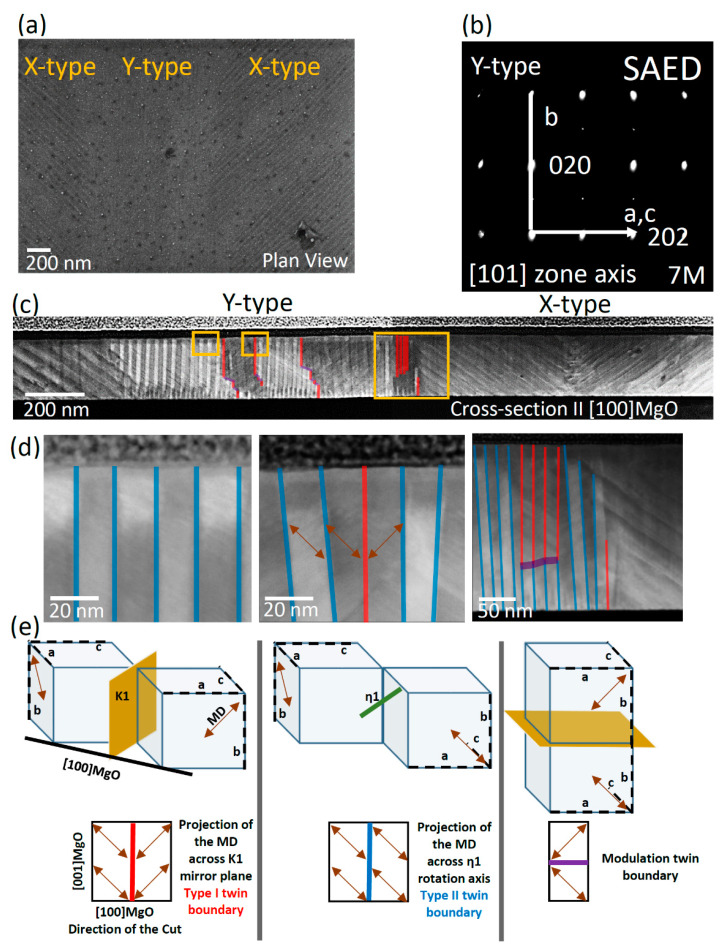
(**a**) SEM secondary electron image of sample #1 showing the coexistence of X-type and Y-type configurations; (**b**) selected-area electron diffraction (SAED) pattern of the Y-type region showing the parameters of the martensitic cell in the Y-type configuration, which have been fitted to 7M monoclinic cell and the orientation of each axis; (**c**) large-scale cross- section high-angle angular dark-field scanning transmission electron microscopy (HAADF) image of the sample; (**d**) magnified HAADF images of the marked areas in panel (**b**) showing the type of twins (red for type I, blue for type II and purple for modulation twin boundaries), the modulation directions are shown by double-headed arrows; (**e**) top: schematic representation of the martensitic cell axis and the modulation direction in Y-type across type I, type II and modulation boundaries (austenitic setting); bottom: the corresponding projection of the modulation direction in the plane of the lamella (FIB-cut along [100]MgO).

**Figure 3:**
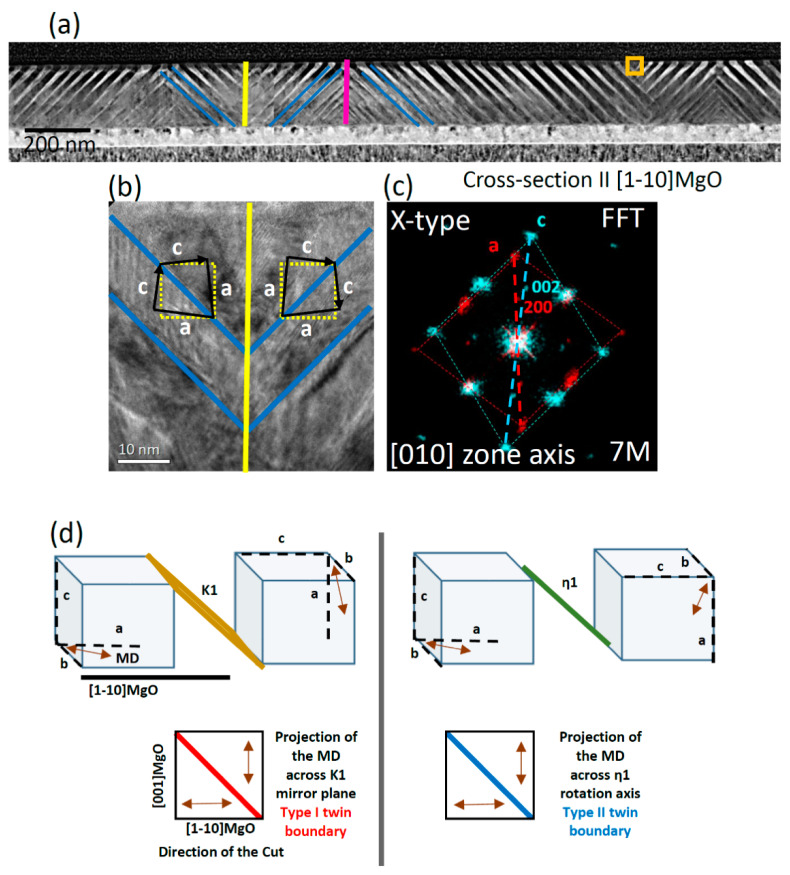
(**a**) Large-scale HAADF image of sample #2, the twin boundaries and the conjugation interfaces are marked by blue, pink, and yellow lines; (**b**) HR-TEM of the marked area in panel (**a**) showing the symmetry of the martensitic cells across the twin boundary and the conjugation interface; (**c**) FFT pattern taken at the twin boundary (shown in panel (**b**), left) showing the a and the c axes (black lines) alternating across the twin boundary, the yellow dashed lines aligned along the [001] and [1−10] directions of MgO are guides for the eyes to evidence the observed cell misorientation; cell parameters are fitted to the 7M monoclinic cell; (**d**) top: schematic representation of the martensitic cell axes and the modulation direction in X-type across type I, type II boundaries (austenitic setting), bottom: the corresponding projection of the modulation direction in the plane of the lamella (FIB-cut along [1−10]MgO).

From the crystallographic point of view, X- and Y-type twin boundaries separate regions following strict twinning relations (Figure 1d). The twinning operators are mirror or (and) rotation. Therefore, based on theoretical models within the continuum theory of martensite, we introduce three atomically sharp crystallographic twin boundaries, which are observed in our epitaxial films (for the crystallographic description, see the Results section):

(1) Type I twin boundary, where the a and c axes of the martensitic cells (austenitic setting) alternate through a mirror plane (K1 is the twinning plane, left panels in Figure 2e and Figure 3d).
(2) Type II twin boundary, in which the a and c axes of the martensitic cells (austenitic setting) alternate by a 180° rotation (around the η1 axis, middle panel in Figure 2e and right panel in Figure 3d).
(3) Modulation twin boundaries (MTB), where only the modulation direction of the martensitic cells alternates across the boundary (right panel in Figure 2e). In this type of boundary, both twinning symmetry relations are satisfied [12,28,29].

Among the twinning systems, type I and type II and their related twinning stress are of special interest, as they alternate the easy magnetization axis in the martensitic cell. 

In the literature about Ni-Mn-Ga, the twinning stress of type II twin boundaries is reported to be up to around twenty times lower than the type I counterpart, i.e., type II twin boundaries are considered highly mobile upon applying external magnetic field [19,30,31]. Therefore, in order to achieve giant magnetic field induced strain, type II twin boundaries are evidently desired. 

In 2015, Yang et al. reported a combination of type I and type II twin boundaries for both X-type and Y-type configurations in 7M Ni-Mn-Ga epitaxial films [25]. In 2017, Niemann et al. reported a model for the X- and Y-type configurations, where combinations of eight compatible twin variants nucleate diamond-like enclosed volumes of martensite. The diamonds grow until they meet each other or (and) meet the substrate. The midribs of the diamonds serve as the twin boundaries (type I, type II, and modulation) [10].

In addition to the above twinning systems, there exist interfaces separating colonies of X- or Y-type twin boundaries in larger scale, for which the exact atomically sharp twinning relations are not fulfilled. These interfaces can be sorted into conjugation interfaces and non-conjugation interfaces, depending on whether they separate colonies of conjugated or non-conjugated planes (Figure 1d) [32,33,34].

Simplified schematic representations of the twin boundaries nucleated from different {101} planes and interfaces connecting the colonies of twin boundaries are provided in Figure 1d. The scheme shows the twin boundaries as the inclined and vertical red lines (X and Y, respectively). The pink line corresponds to a ridge and the yellow line corresponds to a valley [35], both can be categorized as conjugation interfaces. The green lines are assigned for the non-conjugation interfaces [32,33,34]. 

## 3. Results and Discussion

The epitaxial crystallographic relations of the MgO substrate, Cr under layer (for sample #2), and Ni-Mn-Ga cells were determined by TEM analysis as [100]Ni-Mn-Ga//(001)[110]MgO for sample #1 and (001)[100]Ni-Mn-Ga//(001)[100]Cr//(001)[110]MgO for sample #2. The crystal symmetry of the martensitic cells was characterized as sevenfold modulated monoclinic structure [27]. The crystallographic coordinates describing the martensitic cell are provided in Figure 1a. In order to keep the coherency and simplify the description, the martensitic cells will be described only in the austenitic setting (i.e., a, b and c, c is the shortest axis and the easy magnetization axis).

The cell parameters of the martensitic cells were measured by TEM and X-ray diffraction (Figure S3) for sample #1 as a = 0.608 nm, b = 0.578 nm, c = 0.552 nm, γ = 91.5° and for sample #2 as a = 0.609 nm, b = 0.577 nm, c = 0.552 nm, γ = 91.5°.

### 3.1. Symmetry of the Twin Boundaries

To avoid the superposition of the twin boundaries across the TEM lamella, the cross section of the samples were prepared along [100] MgO (for observing Y-type in sample #1) and [1−10] MgO (for investigating X-type in sample #2). 

Figure 2 shows the results obtained for sample #1, presenting the coexistence of X-type and Y-type configurations. The Y-type configuration was induced by a local mechanical stress applied after the growth (Figure 2a) [7]. The lamella for TEM investigation was prepared from the Y-type region. The diffraction pattern taken from the [101] zone axis of the Y-type region is shown in Figure 2b. Considering the orientation of the martensitic cells in the austenitic setting, the b axis of the cells lies out of plane, whereas the a and c axes alternate in the plane of the film (the c axis being the shortest axis and the easy magnetization axis). This is the typical pattern for the Y-type twinning configuration in 7M monoclinic martensitic phase. The HAADF image of the whole lamella is shown in Figure 2c; the three marked square areas are magnified and represented in Figure 2d. As can be seen, inside each variant there are tiny contrast variations with certain directions, which correspond to the 7M contrast modulation of the martensitic phase. The strategy we used to evaluate the type of twin boundaries in Y-type configuration is based on the observation of changes of contrast modulation direction across the boundaries, by HAADF and HR-TEM. This enables us to identify the types of twin boundaries on a large scale. Based on this approach, three types of twin boundaries can be identified, which have been marked with red, blue, and purple lines in Figure 2c,d. The direction of the modulation only changes across the red and purple boundaries. As an example, in the middle HAADF image of Figure 2d, the directions of the contrast variations across the boundaries are shown by double-headed arrows. A higher resolution version of the right image in Figure 2d can be found in the supplementary material (Figure S2a). In addition, in Figure 2e, the schematic explanation of the observed changes is provided. If we consider the martensitic monoclinic cell in the austenitic setting, the direction of the modulation always lies in the plane of the a and b axes (Figure 1b). For the top left scheme of Figure 2e, the K1 mirror plane (type I) serves as the twin boundary by alternating the orientation of the martensitic cell, while for the middle image, the η1 axis (type II) serves as the twin boundary by rotating the cell of about 180°. As for the top right scheme, only the modulation direction (MD) of the cell alternates across the twinning plane. In all the three schemes, double-headed arrows show the MD across the twin boundaries. In the bottom part of Figure 2e, the schemes show the relative (with respect to the top schemes) projection of these MD across the twin boundaries in the plane of the lamella (FIB-cut along [100] MgO), proving the types of twin boundaries in the Y-type configuration of the prepared lamella. We scanned the whole Y-type region to find the distribution of the types of twinning. Only the twin boundaries marked with red and purple in Figure 2c,d were found to have type I and modulation symmetry relations. The rest of the twin boundaries evidently show type II twin boundary. It is worth mentioning that close to the substrate interface (~50 nm), we observed a pronounced branching of the twins with type I boundaries, which are shown in the supplementary material (Figure S2). This represents an experimental evidence for the model proposed as the diamond model of the nucleation and growth of the martensitic nuclei in Y-type configuration [24].

Figure 3a shows the HAADF image of the lamella prepared from sample #2, alternating stripes with bright and dark contrasts, around 45° and −45° tilted with respect to the substrate, which are typical of X-type configuration. 

Besides X-type twin boundaries (marked with blue lines), some conjugation interfaces connecting the colonies of differently inclined twin boundaries at the valley and ridges are marked with yellow and pink, respectively. 

The square marked area in Figure 3a is magnified in HR-TEM (Figure 3b). The corresponding fast Fourier transform (FFT) (Figure 3c), taken at the top left twin boundary of Figure 3b, shows the alternation of the a- and c-axes in the out-of-plane direction; the b-axis always lies in the film plane, which is typically expected for X-type configuration. The FFT reveals a few degrees misorientation of the a- and c-axes with respect to the [001] and [1−10] directions of MgO (dashed yellow lines). In addition, a close inspection of HAADF and HR-TEM images enables us to determine the predominant variant in sample #2. These latter results are consistent with the results obtained by XRD. More details are reported in supplementary material, Figure S3.

For the X-type configuration, due to the orientation of the twin boundaries, as it is shown in Figure 3d, the projections of the resultant MDs for both the mirror and rotation symmetry operators in the plane of the lamella (FIB-cut along [1−10] MgO) are equal. In this case, the presence of the type II twinning system for a cross section along [1−10] MgO is proposed in Ref. [10].

### 3.2. Evolution of the Interfaces

**Figure 4:**
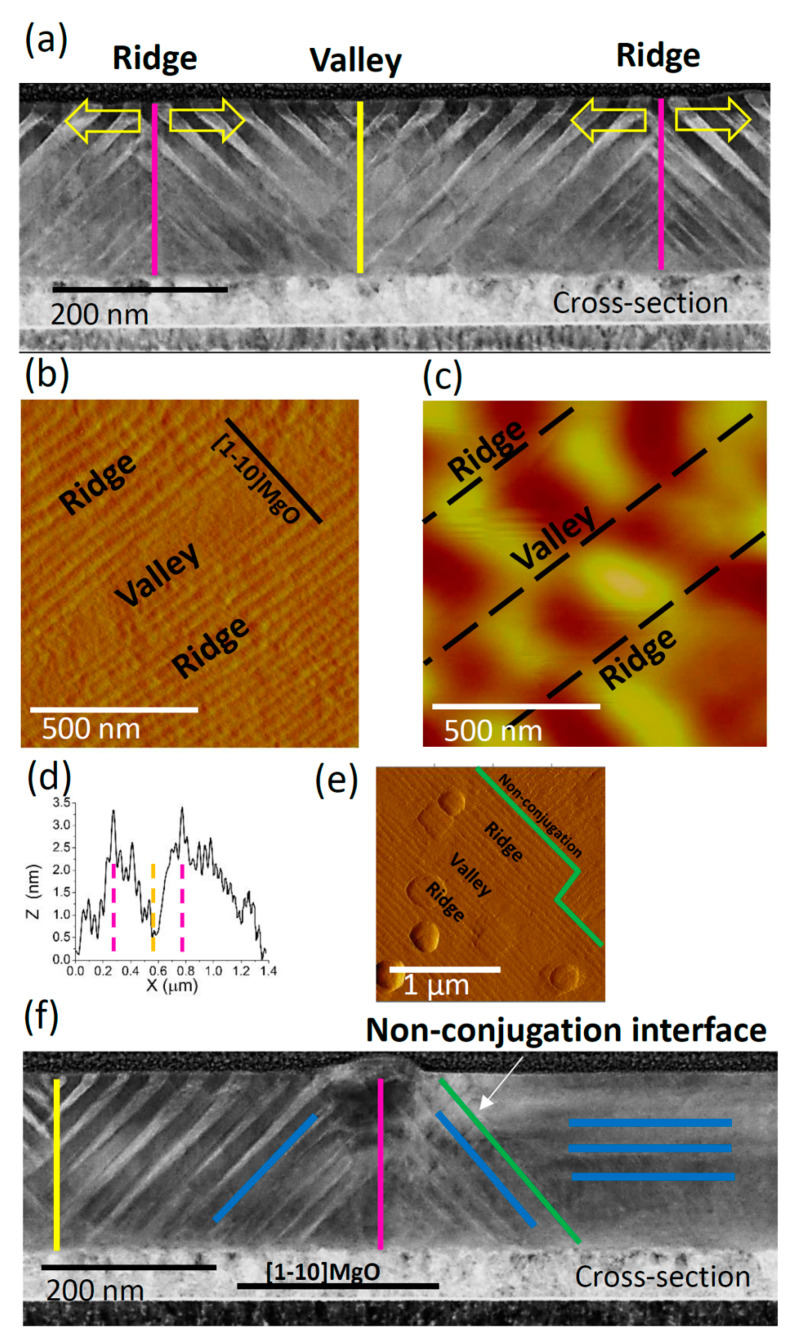
(**a**) HAADF image of sample #2 showing the ridges and valleys created by the conjugation interfaces (pink and yellow lines); (**b**,**c**) AFM/MFM images of sample #2 at RT showing the conjugation interfaces in plan-view (b) and the related disconnections in the magnetic domains (**c**); (**d**) height profile (Z) of the topography (**b**) taken along the [1−10] direction of MgO (X), showing the position of the two ridges and the valley; (**e**) topography image illustrating the conjugation and non-conjugation interfaces (green lines) in plan-view; (**f**) HAADF image of sample #2 showing the non-conjugation interface (green line) in cross section view, the geometry of the twins (blue lines) and the conjugation interfaces (pink and yellow).

Figure 4a shows the HAADF image of conjugation interfaces separating colonies of differently inclined twin boundaries. The conjugation interfaces highlighted in the HAADF image can be directly visualized in the AFM plan view topography shown in Figure 4b. Ridges and valleys are revealed by the height profile of the topography taken along the [1−10] direction of the MgO substrate (Figure 4d). The corresponding MFM image (Figure 4c) shows an inversion of contrast at the conjugation interfaces. The main source of MFM contrast is the variation of the stray field in the direction normal to the sample surface; this reveals that the out-of-plane easy magnetization axis of the martensitic variants inverts the direction across the conjugation interfaces. In order to schematize this effect in the atomic scale we inserted arrows in Figure 3b to evidence both the crystallographic orientations and the magnetization directions (i.e., c axis) across the conjugation interface. 

Figure 4a also shows a variation of the spatial twinning periodicity throughout the film (Ʌ range ≈ 10–30 nm). It is evident that Ʌ decreases close the substrate for the twin boundaries that connect at the ridges, while Ʌ decreases close the surface for the twin boundaries that connect at the valleys. The twin boundaries with low twinning periodicity in the ridge position (pink line) do not reach the surface, while those in the valley position (yellow line) do not reach the Cr underlayer; evidently, because they meet each other before growing through the whole thickness of the film, therefore their further growth is hindered. In addition, more symmetric conjugation interfaces are observed in the lamella at the ridges rather than at the valleys (Figure 3a). Symmetric conjugation typically appears when the two sides of the conjugation system meet at the registry [34]. In the present case (i.e., Ni-Ni-Ga film), it could occur when the twins nucleate at the same point (or grow equally towards each other). All these observations are coherent with in situ AFM topography measurements vs. temperature, and can be explained by a transition route, in which the nucleation of the twin boundaries initiates from the ridges and proceeds until they meet at the valleys, at non-conjugation interfaces or they reach the substrate (Figure 4a). This route will be discussed in detail below.

The other type of martensitic interface, which was shown schematically in Figure 1d, is the non-conjugation interface. An example of this type of interface is shown in the topography plan view (Figure 4e) and HAADF cross section (Figure 4f). They show a blurred region on the right side of the image and a region with well distinguishable twin boundaries on the left side separated by an inclined green line. In the blurred region, the orientations of the twin boundaries are superimposed and parallel to the lamella, causing the observed blurredness. The relative orientations of the twin boundaries are highlighted by blue lines, while the ridge and valley by pink and yellow lines, respectively.

In the martensitic forward and reverse transitions, the interfaces play an important role. In thermoelastic materials, the martensitic forward transition starts with the formation of the phase boundaries and proceeds with moving the phase boundaries, propagating the martensitic phase at the expense of the austenitic phase. The nucleation of the lower symmetry phase is energy costly, a cost which needs to be compensated. To reduce the energy cost, the transition initiates by a heterogeneous nucleation of the low symmetry phase from the most vulnerable regions (e.g., defects, impurities, scratches, etc.). On the other hand, in the reverse transition (martensite to austenite) the material transforms back to the high symmetry austenitic phase [35]. Residuals of untransformed austenitic phase inside the martensitic phase have been suggested to serve as the starting points for the reverse transition. In the literature, the above-described martensitic interfaces are generally suggested to be the regions maintaining the residuals of the untransformed austenitic phase [36]. 

In this work, in order to clarify this point, we explored the X–Y interfaces (Figure 2) and X–X interfaces (Figure 3, both conjugation and non-conjugation) by HR-TEM. No trace of the austenitic phase was found at room temperature by FFT and SAED analysis. 

**Figure 5:**
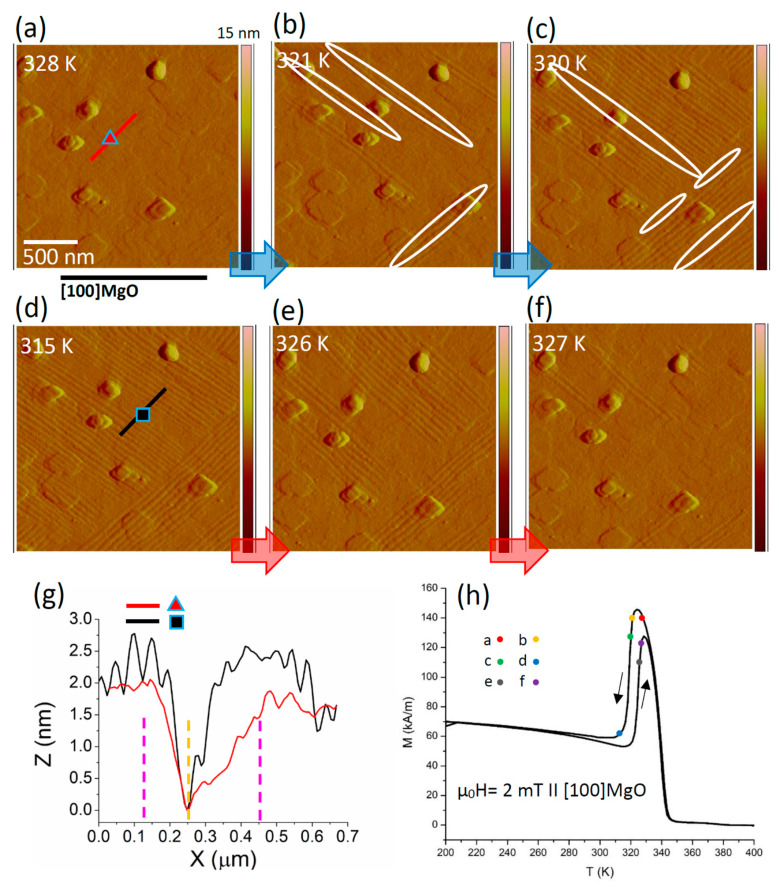
Tracing the formation and annihilation of the interfaces in sample #2 by means of in situ topography imaging vs. temperature. AFM images were captured in different stages of the forward and reverse transition (345K-300K-345K): (**a**) 328K, the film is in the austenitic phase; (**b**) 321K, nucleation of the martensitic phase starting from the ridges (conjugation interfaces), some are highlighted; (**c**) 320K, twin boundaries continue to nucleate and grow till they meet each other, the highlighted valleys and non-conjugation interfaces have not transformed yet; (**d**) 315K, the film is in the martensitic phase, the valleys and the non-conjugation interfaces have transformed as well; (**e**) 326K, the material transforms back to the austenitic phase starting from the valleys and the non-conjugation interfaces; (**f**) 327K, only the ridges are still untransformed; (**g**) height profile of the marked area (the two ridges and the valley are assigned by the pink and yellow dash-lines, respectively); (**h**) initial magnetization vs. temperature curve of the sample with schematic correspondence between the imaging temperatures and the curve.

To deepen the phenomenology of the transition, we measured the evolution of the interfaces (conjugation and non-conjugation) upon phase transition by in situ topography imaging vs. temperature (Figure 5) in sample #2 (all X-type). In X-type microstructure, the pronounced corrugation of surface can be clearly imaged through height contrast by means of atomic force microscopy measurements. In contrast, in the Y-type microstructure, the twin structure can be scarcely visualized by height contrast because of the very small surface corrugation it gives rise to [9]. Sample #2 was cooled down from 345 K to 300 K and subsequently heated up to 345 K, capturing the topography images in different stages of the forward and reverse transition. Figure 5a shows the status of the sample at 328 K (austenitic phase). As the temperature is decreased to 321 K (Figure 5b), the X-type twin boundaries nucleate in isolated regions (ridges, some of them are highlighted), which then continue to nucleate and grow towards each other. The highlighted areas in Figure 5c show the valleys and the non-conjugation interfaces, which are not yet transformed. Upon further decreasing temperature to 315 K (Figure 5d), the scanned area fully transforms to the martensitic phase. 

One can trace the positions of the ridges, valleys, and the non-conjugation interfaces. Upon subsequent heating (to 326 K, Figure 5e), the reverse transition takes place, starting from the positions of the valleys and the non-conjugation interfaces. Figure 5f shows that the ridges degrade at the final stages of the reverse transition. In order to better visualize the positions of the interfaces, the height profile of the marked areas in Figure 5a,d are shown in Figure 5g. As it is shown in Figure 5g, a kind of nanometric surface relief evidently pre-exists close to the martensitic transition. Identifying the origin of this surface relief requires further investigation, taking into account different possibilities, e.g., short-range ordering [37] and the thermomagnetomechanical history of the sample [38]. Finally, for the same sample, the martensitic transition can be followed on the low field magnetization curves over temperature, which are reported in Figure 5h.

With these results, we propose that the nucleation of the martensitic phase in sample #2 starts from the positions of the ridges on the surface of the sample. The twin boundaries continue to nucleate and grow towards each other and towards the substrate, until they meet at the valleys and at the non-conjugation interfaces. However, for the reverse transition the non-conjugation interfaces and valleys transform to the austenitic phase at the initial stages of the transition, while the ridges are the last to transform.

In light of our experiments, we emphasize the primary role played by the interfaces in the transition route, and we can deny that it has to be related to residuals of the austenitic phase.

Ni-Mn-Ga cells on the surface of the film provide an additional degree of freedom compared to the cells at the interface with the substrate, which impose huge pinning constraint [39]. The surface of the film serves as the interface for the heat exchange, facilitating the transition [40]. Therefore, upon the forward transition, it is likely that the heat exchange favors the nucleation of the martensitic phase primarily on the surface and at the ridges having convex shape. 

In addition, these coarse corrugations break the elastic homogeneity of the material, facilitating the heterogeneous nucleation of the martensitic phase. Upon the nucleation of the martensitic twin boundaries and their subsequent growth in the austenitic matrix, the created elastic strain energy typically dissipates in the form of heat and acoustic waves and partially stores in the material in the form of stored elastic energy. This energy is related to the irreversible steps of the transition, e.g., the obstacles stopping the growth, therefore it is thermodynamically irreversible [41]. The stored elastic energy works like a spring by partially storing the strain energy during the forward transition. Over the reverse transition, the stored energy is released back serving as a driving force; therefore, it could be mechanically reversible. 

For a multivariant system, where the self-accommodation of the twin variants takes place by the coalescence of differently oriented equivalent boundaries, the dissipation and storage of the elastic strain typically occurs locally upon the nucleation as well as at pinning obstacles [41]. This makes conjugation and non-conjugation interfaces the critical regions of the sample (Figure 4). 

The size of twin boundaries is expected to be directly proportional to the dissipated energy over the martensitic transition [22]. Thus, the stored elastic energy is expected to be inversely proportional the size of the twin boundaries. The low-twinning periodicity boundaries in Figure 4a,f, which do not grow across the whole thickness of the film, are expected to have a larger portion of stored elastic energy. 

The stored elastic energy also regulates the sequence of the transition of the twin boundaries: in Figure 5, the first twin boundaries that appear at the forward transition are the last boundaries to disappear at the reverse transition, which is in agreement with the model proposed in [41,42] for thermoelastic martensites. In fact, the boundaries that appear first over the forward transition are typically the largest, which dissipate the largest energy in the form of heat and acoustic waves and store the lowest elastic energy. On the contrary, the boundaries that appear as the last over the forward transition are typically the smallest, which dissipate the lowest energy in the form of heat and acoustic waves and store the highest portion of elastic energy. These smaller boundaries annihilate first over the reverse transition (compare Figure 4 and Figure 5). 

Recently, the transition temperature of a 400 nm epitaxial Ni-Mn-Ga film was investigated by a nanolocalized scanning thermal microscopy, reporting a considerable temperature gradient (~20 K) for the reverse phase transition of different regions of the sample [43]. Based on the route provided in this study, the reported temperature gradient is likely to be linked also to the transition temperature difference for the martensitic interfaces (i.e., ridges and valleys).

In a system where the transition involves the nucleation and growth of a ferromagnetic phase in a paramagnetic matrix, such as our Ni-Mn-Ga thin film, surface topography and spatial distribution of nucleation sites may have an important influence on the magnetostatic energy. A quantitative evaluation in the specific case would require an accurate modeling, such as proposed for bulk La-Fe-Si [44], a system for which the preferential nucleation of the ferromagnetic phase at the convex areas has been experimentally demonstrated [40].

Our investigation provides a direct evidence of the major role played by the martensitic interfaces on the forward and the reverse transition in epitaxial Ni-Mn-Ga films (Figure 5). We propose that the forward transition initiates heterogeneously from the position of the conjugation interfaces, i.e., ridges on the surface of the film. The twin boundaries continue to nucleate and grow until they meet at the conjugation interfaces, i.e., valleys and the non-conjugation interfaces, where the growth is hindered. In these regions, the elastic strain energy created during the transition is partially stored. The stored energy in these regions serves as the driving force for the reverse transition by initiating the nucleation of the austenitic phase.

## 4. Conclusions

We investigated the crystallographic relations between the twin boundaries and interfaces in Ni-Mn-Ga epitaxial films from the atomic scale to the microscale and accompanied this investigation with the direct observation of the evolution of the martensitic interfaces vs. temperature. 

Based on the symmetry relations between the twin variants, we identified the types of twin boundaries and the twinning interfaces, i.e., ridges, valleys, and non-conjugation interfaces. 

Using the change of modulation direction across the boundary observed by HAAFD and HR-TEM, in Y-type regions we were able to determine the presence of type I, type II, and modulation twin boundaries with a dominant presence of type II. 

Beyond these findings, we propose a transition route originating from the martensitic configuration, highlighting the major role played by the different martensitic interfaces. The forward transition starts with the heterogeneous formation of twin boundaries at the position of the ridges on the surface of the film and moves towards the substrate. The twin boundaries continue to nucleate and grow until they meet at the other kind of conjugation interfaces, i.e., valleys, or at non-conjugation interfaces, where the growth is hindered. In these regions, the elastic strain energy created during the transition is partially stored. This stored energy serves as the driving force for the reverse transition by initiating the nucleation of the austenitic phase. 

In conclusion, this paper sheds light into the direct link between the martensitic configuration at the different length scales and the martensitic forward and reverse transitions. The present results represent a step forward in the understanding of the transition processes and pave the way to the possibility of tuning the characteristics of the transition, e.g., hysteresis and transition width, by microstructural engineering aimed at the full exploitation of martensitic Heuslers in cyclic applications.

## Supplementary Materials

The following are available online at https://www.mdpi.com/1996-1944/13/9/2103/s1, Figure S1: (a,b) atomic force microscopy images and (c,d) magnetic force microscopy images at room temperature showing the typical X-type microstructure for the as-grown sample #1 and #2, Figure S2: Type of twin boundaries in the Y-type configuration of the lamella prepared out of sample #1: (a) HAADF image of the lamella, examples of different types of twins are highlighted. In addition, the observed branching of the Y-type twins close to the MgO interface is highlighted, (b) HR-TEM of an example of the type I twin boundaries alternating the modulation direction of the cells across the boundaries near the substrate and Figure S3: X-ray diffraction of sample #2 at 223 K, the dark blue shows the {h00} family of epitaxial peaks in a normal theta-2theta scan for 2theta = 58–70° and the purple shows the asymmetric scan (2° offset). The (400) and (004) martensitic peaks measured in the asymmetric scan are assigned for the a and c-axis of the martensitic cells out-of-plane of the film, respectively. The relative intensity counts of the two peaks is reported in the figure. Considering the possible errors of the measurement and the calculations, the values are in reasonable agreement with the relative intensity counts calculated from the STEM image of the lamella #2 for a and c-axis out-of-plane of the film (34% a-axis and 66% c-axis) As it was explained in the manuscript, the peaks related to the out-of-plane a and c-axis typically appear with slight misorientation with respect to the substrate normal plane due to the slight (CW or CCW) rotation of the martensitic cells around the b axis upon the formation.
