# Supplementary material for: Following the Martensitic Configuration Footprints in the Transition Route of Ni-Mn-Ga Magnetic Shape Memory Films: Insight into the Role of Twin Boundaries and Interfaces
Source: Materials (Basel). 2020 May 1;13(9):2103. doi: 10.3390/ma13092103 (PMC7254361; doi:10.3390/ma13092103)
Supplement: Supplementary file 1 [file materials-13-02103-s001.pdf]

Supplementary Materials

Following the Martensitic Configuration Footprints in the Transition Route of Ni-Mn-Ga Magnetic Shape Memory Films: Insight into the Role of Twin Boundaries and Interfaces

Milad Takhsha Ghahfarokhi, Lucia Nasi, Francesca Casoli, Simone Fabbrici, Giovanna Trevisi, Riccardo Cabassi and Franca Albertini *

Institute of Materials for Electronics and Magnetism, National Research Council (IMEM-CNR), Parco Area delle Scienze 37/A, Parma 43124, Italy; milad.takhsha@imem.cnr.it (M.T.G.); lucia.nasi@imem.cnr.it (L.N.); francesca.casoli@imem.cnr.it (F.C.); simone.fabbrici@imem.cnr.it (S.F.); giovanna.trevisi@imem.cnr.it (G.T.); riccardo.cabassi@imem.cnr.it (R.C.)

* Correspondence: franca.albertini@imem.cnr.it

Received: 22 March 2020; Accepted: 28 April 2020; Published: date

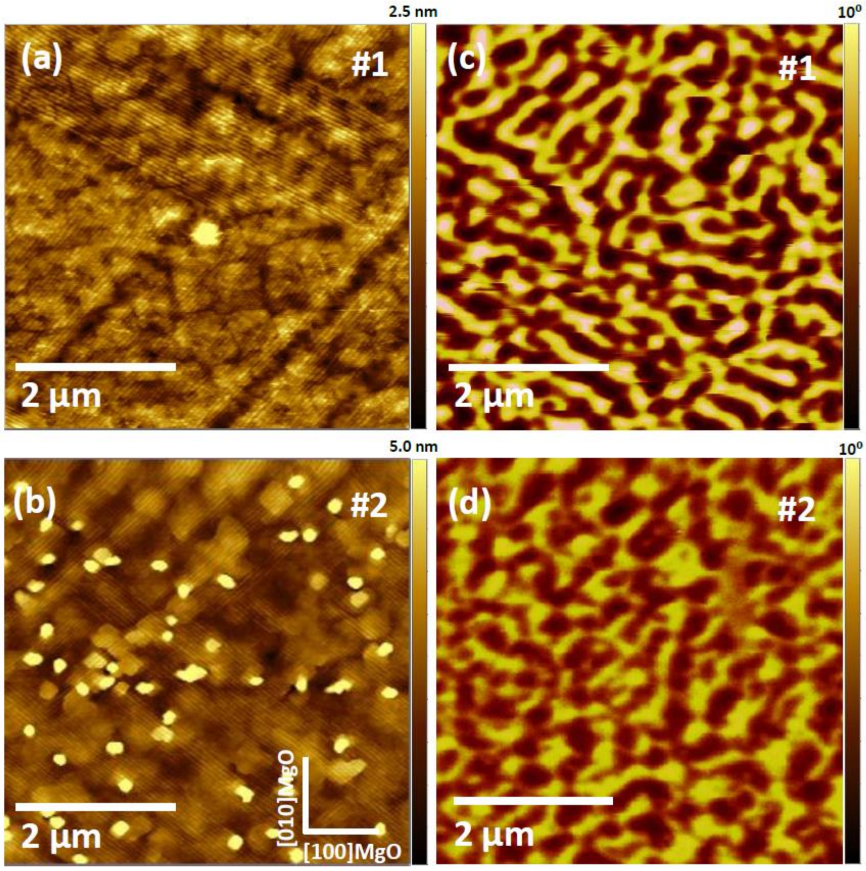

Figure S1. (**a**,**b**) Atomic force microscopy images and (**c**,**d**) magnetic force microscopy images at room temperature showing the typical X-type microstructure for the as-grown samples #1 and #2.

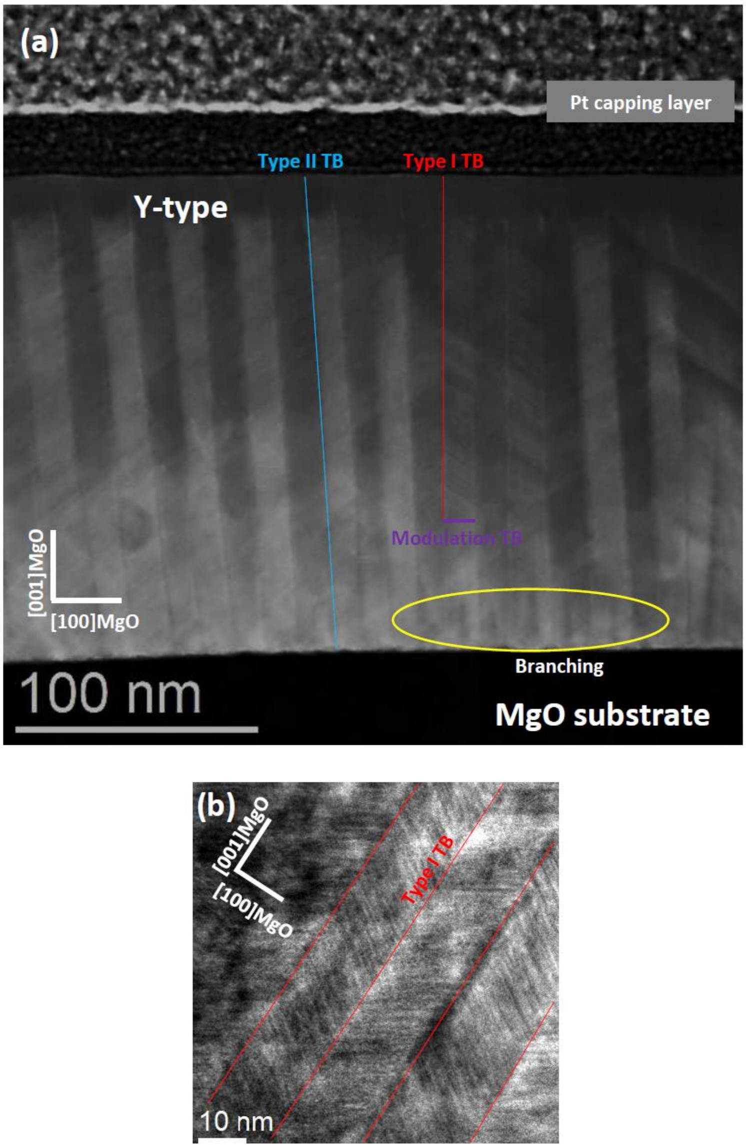

Figure S2. Type of twin boundaries in the Y-type configuration of the lamella prepared out of sample #1: (a) High-angle angular dark-field scanning transmission electron microscopy (HAADF) image of the lamella, examples of different types of twins are highlighted. In addition, the observed branching of the Y-type twins close to the MgO interface is highlighted, (b) HR-TEM of an example of the type I twin boundaries alternating the modulation direction of the cells across the boundaries near the substrate.

The FFT pattern (Figure 3c) shows ~2.6° clockwise (CW) misorientation for c axis and ~2.0° counterclockwise (CCW) misorientation for a axis with respect to the growth direction [001] MgO. This angular deviation has also been reported for Ni-Mn-Ga single crystals by a few degrees rotation around the b axis (CW and CCW) with respect to the origin to reduce the strain cohesion at the phase boundary [1]. This angular deviation is only evident for X-type configuration in the cross-sectional view, as the b axis of the martensitic cells lies in the plane of the film, perpendicular to the direction of the lamella cut. The observed misorientation is consistent with the X-ray diffraction analysis reported in Figure S3, i.e., typically the diffraction peaks of (400) and (004), corresponding to the out-of-plane a and c axes of the martensitic cells only appear by the asymmetric scan (around 2° offset).

In addition, in the HAADF image (Figure 3a), the regions with c axis of the martensitic cells out-of-plane of the film correspond to the dark stripes and the regions with a axis out-of-plane of the film correspond to the bright stripes. The relative distribution of the contrasts along the central part of the lamella shows ~66% counts for the dark and ~34% counts for the bright contrast, which in good

agreement with the relative intensity calculated for of the diffraction peaks corresponding to c axis out-of-plane and a axis out-of-plane (Figure S3).

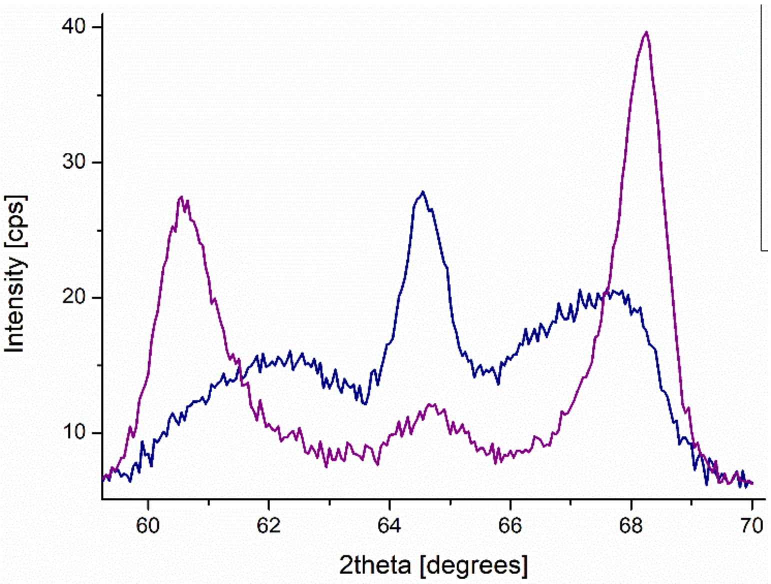

Figure S3. X-ray diffraction of sample #2 at 223 K, the dark blue shows the {h00} family of epitaxial peaks in a normal theta-2theta scan for 2theta = 58–70° and the purple shows the asymmetric scan (2° offset). The (400) and (004) martensitic peaks measured in the asymmetric scan are assigned for the a and c axis of the martensitic cells out-of-plane of the film, respectively. The relative intensity counts of the two peaks is reported in the figure. Considering the possible errors of the measurement and the calculations, the values are in reasonable agreement with the relative intensity counts calculated from the STEM image of the lamella #2 for a and c axis out-of-plane of the film (34% a axis and 66% c axis) As it was explained in the manuscript, the peaks related to the out-of-plane a and c axis typically appear with slight misorientation with respect to the substrate normal plane due to the slight (CW or CCW) rotation of the martensitic cells around the b axis upon the formation.

References

Chulist, R., Czaja, P., Tokarski, T., Kuksgauzen, I.; Chumlyakov, Y.I. Orthogonal shear process in Ni-Mn-Sn single crystal. Int. J. Plasticity **2019**, 114, 63–71.
